# Army News

**Published:** 1918-10

**Authors:** 


### Army News.

Captain Manton M. Carrick, formerly of Dallas, but now of the Medical corps of the United States Army, stationed at Fort Oglethorpe, Ga., fell and fractured his right forearm, and as a result, is confined in the hospital. Dr. O. J. Cook of Dallas, now also a Medical Officer in the Army, likewise is a patient in the hospital, having just been operated upon for appendicitis.

Lieutenant T. C. Brewer of Fort Worth, who volunteered some time ago, has been ordered to Camp Travis, where he is now stationed at the base hospital.

Dr. Anna L. Van Sholly, a member of the staff of the Women's Overseas hospital, and now doing service in France, has been awarded the croix de guerre and has been commissioned as a lieutenant in the French army by that government for her valuable services at the front. The hospital of which she is a member was organized by the Woman's Suffrage party.

In order to provide medical attention for the members of the Students' Army Training Corps at the University, two additions have been made to the medical force of that institution in the persons of Dr. S. N. Key and Dr. George M. Decherd. These two physicians, together with Dr. Joe Gilbert, the regular medical officer of the University, will have offices on the first floor of the main building.

Mrs. T. S. Williams, Dallas, Texas, Superintendent of nurses at Parkland Hospital, is in receipt of a cablegram from her husband, who has been in a British army hospital for several months after having been gassed in France, to the effect that he is sailing for America. Dr. Williams joined the British forces more than a year ago as a member of the Medical Corps.

Dr. Kent V. Kibbie, Fort Worth physician, has been commissioned a captain in the Medical Corps of the Army. He will leave in a short time for the Medical Officers training camp at Fort Oglethorpe, Ga.

Dr. Kibbie was, for several years, professor of anatomy at the Texas Christian University Medical College.

"The French people have a spirit which is unbreakable. This is a wonderful country," writes Major James Hal Gambrell to friends in Dallas.

Dr. Gambrell, who for some years, was a practicing physician in Dallas, is now with the U. S. Medical Corps in France. He writes that the American soldiers do not know the meaning of fear and refuse to see danger to themselves. He says that they have an insatiate curiosity about regarding everything which is new to them.

Dr. A. L. Ridings,physician of Sherman, has been appointed a captain the Army Medical Corps.

Promotion to major of Captain Arthur J. Boyd, commandant of the Post Hospital at Taliaferro Field, was announced, Friday.

Major Boyd is a native of Paris, Texas, and a graduate of the Kentucky School of Medicine, Louisville, Ky. After graduation from the Medical School he was interne at St. Joseph's Hospital, Louisville, for two years and was then seventeen years head of the department of abdominal surgery at his alma mater.

His home immediately before the war was in San Angelo. He was commissioned a captain on October 1, 1917, and assigned to active duty January 9, 1918, when he came to Taliaferro Field Hospital as the head of the department of surgery. He was later hospital adjutant and was made commandant of the hospital upon the departure of Major Coumbe a few months ago.

Dr. C. T. Steen of Dawson, who recently received an appointment as First Lieutenant in the U. S. Medical Department, is in Fort Riley, Kansas, where he will enter training.

Dr. Thomas E. Cook of Belton, has been commissioned First Lieutenant in the Medical Reserve Corps, and upon receipt of his commission left at once for Fort Oklethorpe, Ga.

Dr. A. C. Rogers of Odell, was recently commissioned First Lieutenant in the Medical Reserve Corps.

Dr. R. F. Miller, formerly a medical practitioner of Sherman, has arrived in France. He is at one of the base hospitals and has been given the rank of Captain.

Lieutenant Hester B. Smith, who has been at Camp Bowie for several months, has been transferred to Camp Logan, Houston, Texas. He will be assistant to the camp surgeon. He is a well known Dallas physician, who retired from his practice to engage in Government work.

Dr. T. D. Roundtree of Rockdale, has received his commission and is now a full-fledged Major, Medical Corps, 10th Infantry, Texas National Guard

Dr. Taylor C. Gilbert, of Dallas, recently commissioned a First Lieutenant in the Medical Reserve Corps, has gone to Camp Bowie, where he will be assigned to the base hospital. Dr. Gilbert was County Health Officer.

---

THE red color on a woman's cheek may be due to one of three causes; a flush, a blush, or a brush.—J. F. S.

